# A Comprehensive Study on Enhancing Microbicidal Activity of Pure and Ion-Exchanged Zeolites Through Structural and Chemical Determinants

**DOI:** 10.1049/nbt2/7012728

**Published:** 2025-09-27

**Authors:** Hadi Tabesh, Shabnam Kharrazi, Mostafa Bashiri Barazandeh, Parastoo Ebadoulah Poursafa, Ali Poorkhalil

**Affiliations:** Department of Biomedical Engineering, School of Life Science Engineering, College of Interdisciplinary Science and Technologies, University of Tehran, Tehran, Iran

**Keywords:** antifungal, antimicrobial, ion-exchange, metallic ions, zeolite

## Abstract

Zeolites are crystalline aluminosilicate materials known for their unique structures and small pores, making them highly suitable for various applications, including antimicrobial uses. Their porous surfaces enable them to act as carriers for metal ions, enhancing their antibacterial potential. A recent comprehensive review of the literature assessed the antibacterial activity of both natural and synthetic zeolites, with a specific focus on their performance after being modified with metal ions. The study confirmed that while unmodified zeolites possess some inherent antibacterial properties, their effectiveness is generally limited to high concentrations. In contrast, zeolites modified with metal ions, such as silver (Ag), copper (Cu), or zinc (Zn), demonstrate significantly enhanced antimicrobial effects at much lower concentrations. Among the metal-modified zeolites, Ag-treated zeolite A (ZA) emerged as the most effective, exhibiting a remarkably low minimum inhibitory concentration (MIC) of just 16 µg/mL against various bacterial strains. This heightened activity is attributed to the controlled release of Ag ions and the high ion-exchange capacity of ZA, which allows for sustained antimicrobial action. These findings suggest that metal-exchanged zeolites, particularly those with high ion-retention capabilities, hold strong potential as long-lasting and efficient antimicrobial agents. Such materials could be valuable in medical, environmental, and industrial applications, especially where bacterial resistance is a growing concern.

## 1. Introduction

Zeolite is derived from the Greek words “zeo” and “lithos,” meaning “to boil” and “stone,” respectively, was first discovered almost 250 years ago [1–4]. Zeolites are aluminosilicates with tetrahedral framework structures based on aluminate (AlO_4_) and silicate (SiO_4_) [5–8]. Essentially, aluminum substitutes for silicon (Al^3+^ ↔ Si^4+^) in the pure silica frameworks, resulting in an overall negative charge that is typically balanced by extra-framework cations within the cavities [9, 10].

This negative framework charge is the fundamental reason behind the ion-exchange ability of zeolites. The extra-framework cations (e.g., Na^+^, K^+^, Ca^2+^) are not part of the rigid lattice and can be readily exchanged with other cations from the surrounding media without altering the structural integrity of the zeolite. This property allows zeolites to selectively adsorb and release metal ions, making them valuable for applications in catalysis, environmental remediation, and antibacterial treatment [11–13]. A simplified empirical formula for an aluminosilicate zeolite is M*_x_^n^*^+^/_*n*_ Al_*x*_Si_1_₋_*x*_O_2_•*yX*, where M^*n*+^ represents organic or inorganic cations (e.g., Na^+^), and *X* denotes adsorbed species (e.g., H_2_O) [14].

Zeolites possess unique properties that make them suitable for various applications, such as heterogeneous catalysis, ion exchange, biosorption supports, adsorption, and polymer catalytic degradation [15–17]. They can also serve as effective antibacterial agents, especially when the extra-framework cations are exchanged with metallic cations exhibiting high antibacterial activities [18–20]. Copper (Cu), silver (Ag), and zinc (Zn) ions have been extensively studied as antibacterial agents in ion-exchanged zeolites [18, 21, 22]. The ion-exchange capacity in zeolites is related to the silica/alumina (Si/Al) ratio in their structures, with zeolites having low Si/Al ratios (~1–1.5) demonstrating higher ion-exchange capacities compared to other zeolites [23–25].

Despite numerous studies investigating the antibacterial properties of ion-exchanged zeolites, our extensive literature review revealed a lack of publications discussing the influencing parameters for optimizing the microbicidal properties of zeolites. Consequently, the relationships between the type of zeolite, Si/Al ratio, metallic ion release rate, and antibacterial properties of pure and ion-exchanged zeolites remain ambiguous.

Zeolites are widely used in various industrial applications due to their highly adjustable physicochemical properties, making them a significant focus of research in recent years. These materials can be sourced from natural deposits or synthesized from high-silica and high-aluminum raw materials. However, natural zeolites often exhibit inferior performance compared to synthetic versions because of complex formation conditions, unstable chemical compositions, and impurities. This limitation has led to an increasing demand for synthetic alternatives in industrial applications. Recently, several types of solid waste, including coal fly ash, coal gangue, rice husk, sugarcane bagasse, and coal gasification slag, have been explored as sources of silica and alumina for zeolite synthesis. These solid wastes are advantageous due to their abundant availability and low cost, making them promising raw materials for producing synthetic zeolites [25–30].

This article aims to analyze the most current literature addressing the antibacterial activities of pure and ion-exchange zeolites. By compiling and comparing reported outcomes from selected articles, we seek to identify the key factors influencing the antibacterial effectiveness of zeolites based on the type of zeolites, microorganisms, and experimental conditions. The significant microbicidal results of pure and ion-exchanged zeolites against tested bacteria and fungi will be highlighted in this article.

## 2. Zeolite Types

**Figure 1:**
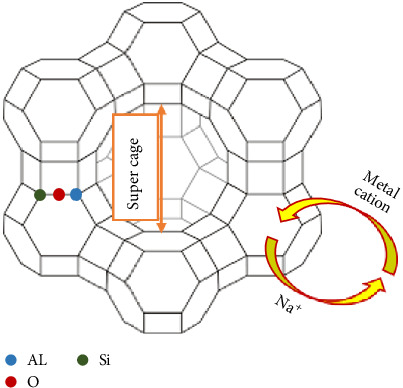
Schematic representation of the basic structural framework of zeolites.

Zeolites consist of over 50 different minerals, comprising silicon and aluminum, each with diverse physical and chemical characteristics. The zeolite structure is characterized as a network of TO_4_ tetrahedral units (T:Al and Si) covalently bonded to four oxygen atoms, enclosing a silicon or aluminum cation. The crystal structures of zeolites are built from primary building units (PBUs) consisting of (AlO_4_)^5−^ and (SiO_4_)^4−^ tetrahedra and secondary building units (SBUs) formed by combining PBUs with adjacent tetrahedra through shared oxygen. SBUs can take the form of single rings, double rings, and polyhedral structures, allowing for the creation of unique cage architectures through various linkages [26, 27]. This network contains channels and cages [17–21, 23–28, 31], as illustrated in Figure 1. Typically, these open channels and cages are occupied by extra-framework metal ions and H_2_O molecules [29].

### 2.1. Natural Zeolites

To date, around 45 different types of natural zeolites have been discovered, with clinoptilolite, analcime, chabazite, laumontite, mordenite, and philistine being among the most prevalent. However, clinoptilolite and mordenite, both containing ~70% silica, are extensively utilized, for instance, as water filters and odor control agents [30, 32, 33].

Clinoptilolite zeolite (CZ), a significant natural zeolite found in numerous deposits worldwide, is a microporous crystal containing alkaline earth and alkali cations. It exhibits a porosity of ~34% and an ion-exchange potential of up to 2.3 meq/g, with a Si/Al ratio varying between 4.0 and 5.7 [34–37].

Natural zeolites are primarily of hydrothermal and volcanic origin. They can be found in crystallized forms within igneous and metamorphic rocks, as well as in smaller granular forms accumulated in sedimentary rocks. Ocean floor sediments are abundant and rich in zeolites, though these deposits remain largely inaccessible to humans. Nevertheless, these minerals can also be significant components of tuffs or clay. The surface retention of zeolite sediments enables relatively simple extraction through opencast mining, making them highly suitable for broader applications. It is worth noting that the naturally occurring zeolites with practical significance include clinoptilolite, mordenite, and chabazite [21, 38–48].

### 2.2. Synthetic Zeolites

While zeolites are naturally occurring minerals, there are also various types of synthetic zeolites. Among them are zeolites with a low Si/Al ratio [45, 46]. For example, ZSM-5 (ZS) has an MFI (ZS-type) framework with a Supercage of 4.5–6 Å [24–30, 32–47], exhibiting stronger adsorption capacities due to high polarity. There are notable differences between natural and synthetic zeolites. Synthetic zeolites have a significantly enhanced ability to adsorb heavy metal ions (e.g., Pb^2+^, Cu^2+^, Cd^2+^, Cr^3+^, Zn^2+^, and Ni^2+^) compared to natural zeolites [49–51]. Furthermore, in comparison with natural zeolites, synthetic zeolites have a larger supercage, which enables the adsorption of larger molecules and expands the range of potential applications. The aluminum content in these zeolites can be adjusted and modified during the synthesis process [52–55].

There are ~130 synthetic zeolites, including zeolite A (ZA), zeolite Y (ZY), zeolite X (ZX), ZS zeolite, etc. [27–30, 32–38, 56]. ZA, for instance, is widely used in industrial processes such as ion exchange, petrochemical cracking, solvent separation, and gas and pollutant removal [39–42]. It has a Si/Al ratio of about 1 and exhibits excellent ion exchange capacity [24]. ZY, synthesized in 1964, has a Si/Al ratio ranging from 1.5 to 3.8 and a framework topology similar to that of ZX [39, 43, 44]. ZS, with the capability to be synthesized with various Si/Al ratios greater than 1, is known for its catalytic versatility [39]. Faujasite zeolite (FZ), a zeolite with the Faujasite (FAU) topology, has a Si/Al ratio ranging from 4.5 to 6.0 [24]. This ratio can be adjusted between 1 and 3 through hydrothermal synthesis or greater than 3 through postsynthetic modifications, making it widely applicable in adsorption and catalyst applications [44].

Despite differences in structure and composition, these synthetic zeolites share several key characteristics, including tunable Si/Al ratios, well-defined microporous frameworks, and high ion-exchange capacities, which contribute to their effectiveness in adsorption, catalysis, and separation processes [57, 58].

Table 1 classifies the most applicable natural and synthetic zeolites used in antimicrobial investigations, along with their physical properties and chemical formulas.

Over the past decade, there has been an increasing focus on sustainable green development by national governments, coupled with rapid advancements in the field of microporous materials. This has resulted in the publication of numerous high-quality studies and reviews on zeolites. Since 2002, various reviews have examined the synthesis of zeolites from different waste materials, such as fly ash and biomass ash, each explored individually. The synthesis methods and applications of these zeolites show notable similarities. In recent years, significant progress has been made in research on synthesizing zeolites from a broader range of industrial and agricultural solid wastes. The sources of these solid wastes have expanded beyond fly ash to include gangue, biomass, gasification slag, and municipal solid waste. Moreover, synthetic zeolites have been modified for various applications, such as pollutant adsorption in wastewater treatment, atmospheric purification, agriculture, and industrial catalysis. However, a comprehensive review focusing specifically on the synthesis of zeolites from solid wastes is still lacking [64–73].

## 3. Microorganism Types

Microbes, also known as microorganisms, are microscopic organisms that are widely distributed in the natural world. They can exist as organized clusters, multicellular organisms, or single-celled entities. From causing illnesses to offering advantages in food production, biotechnology, and medical diagnostics, microbes have a wide range of functions in the environment and in human life [74–76]. Serious health problems like meningitis, diabetic foot infections, and sepsis can result from microbial infections [48, 77]. Nonetheless, the majority of studies concentrate on examining zeolites' antimicrobial capabilities against bacteria and fungi.

Each group has distinctive structural and functional features. Archaea are prokaryotes; they resemble bacteria in many ways but differ from them in membrane composition and genetic sequences [78, 79]. They tend to live in extreme environments. Protozoa are typically unicellular eukaryotes that often function as parasites [80]. Algae are photosynthetic eukaryotes, either unicellular or multicellular, that are major components of aquatic ecosystems [81]. Viruses are acellular entities that must infect host cells and can only replicate inside those cells but can infect any type of organism [82, 83]. Although all microbial groups are valuable, most antimicrobial studies focus on bacteria and fungi, especially with zeolites.

Bacteria are prokaryotic microorganisms characterized by the absence of a true nucleus and membrane-bound organelles. Typically, they are classified as Gram-positive or Gram-negative, based on differences in their cell wall structure and their reaction to the Gram staining procedure. Gram-positive bacteria possess a notably thick peptidoglycan layer, which effectively retains the crystal violet stain during this process and results in a distinct coloration under microscopic examination. In contrast, Gram-negative bacteria feature a comparatively thinner peptidoglycan layer and an additional outer membrane comprised of lipopolysaccharides. These structural distinctions are clinically significant, as the presence of the outer membrane in Gram-negative bacteria tends to confer greater resistance to many antimicrobial agents, posing challenges for treatment and infection control [84–87].

Fungi are heterotrophic, eukaryotic, nonvascular organisms that are unable to perform photosynthesis because they lack chlorophyll. They reproduce by producing spores and can be found as filamentous molds or single-celled yeasts. Commonly found in the environment, fungi can cause opportunistic infections, especially in people with weakened immune systems. They are important topics for antimicrobial research because of their unique biochemical processes and complex cellular structures [88, 89].

The increasing concern over microbial pathogenicity and rising antimicrobial resistance underscores the urgent need for novel antimicrobial materials. Zeolites, with their unique porous structures and ion-exchange capabilities, have demonstrated promising antimicrobial activity against both bacteria and fungi. This makes them attractive candidates for various biomedical and environmental applications [90, 91].

Table 2 classifies and abbreviates the microorganisms primarily used in antibacterial and antifungal investigations of pure and ion-exchanged zeolites.

## 4. Antibacterial and Antifungal Investigation Techniques

In research involving zeolites, various methods are utilized to evaluate antibacterial activity, including agar disc diffusion, time-kill tests, dilution methods, flow cytometry, ATP bioluminescence assays, and bioautography-thin layer chromatography (B-TLC). However, dilution and diffusion methods have been more commonly employed for direct evaluation.

### 4.1. Dilution Method

**Figure 2:**
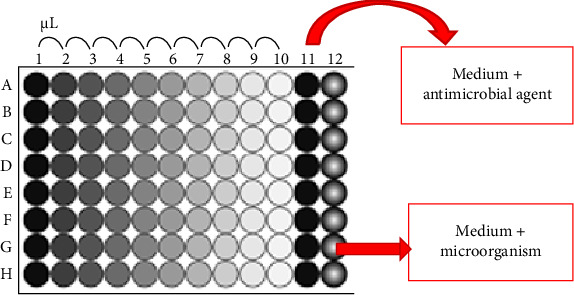
A schematic diagram of an antibacterial investigation technique based on the dilution method.

The dilution method is employed to assess the minimum inhibitory concentrations (MICs) following the guidelines provided by CLSI and the European Committee on Antimicrobial Susceptibility Testing (EUCAST) [92]. This method involves growing bacteria in a liquid medium (Mueller–Hinton Broth) to a 0.5 McFarland standard (1 × 10^7^ colony-forming unit [CFU]/mL) for 24 h. Subsequently, a volume of 100 µL of the microbial suspension is introduced into each well of a 96-well plate. Then, 100 µL of the antimicrobial agent is added to well number 1; after thorough mixing, 100 µL from well number 1 is transferred to well number 2 [93]. This process is repeated for each subsequent well until the last one, with the excess 100 µL from the last well discarded. Finally, 15 µL of the test microorganism is introduced into each well, and the plates are subsequently incubated for 16–20 h to determine the MIC by observing any discoloration [94–97].  Figure 2 illustrates a simplified diagram of the dilution method.

### 4.2. Diffusion Method

**Figure 3:**
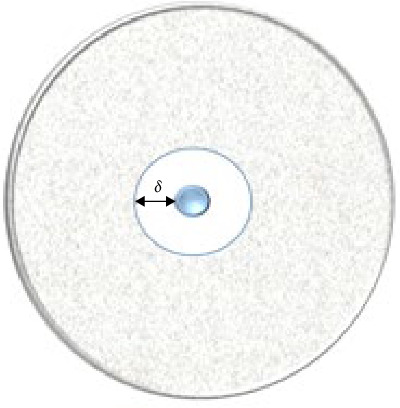
A simplified diagram of antibacterial investigation result based on the disc diffusion method.

The disc diffusion method is a widely used technique for investigating antibacterial properties [98]. This method involves using Mueller–Hinton agar-coated plates that are then incubated at temperatures ranging from 30 to 37°C. To begin, four or five colonies from a pure bacterial culture are transferred with a wire loop to 5 mL of 0.9% NaCl solution. The bacterial concentration is then allowed to reach 0.5 McFarland. After the bacteria have grown in the 0.9% NaCl solution, a sterile cotton swab is immersed in the bacterial suspension and utilized to inoculate the agar by streaking it with the inoculum. The plates are then rotated by 90°, and the process is repeated. Next, a disc containing the antimicrobial agent being tested (typically with a 6 mm diameter) is placed on the surface of the inoculated bacteria, and the plates are subsequently incubated at 37°C for 16–18 h to observe the zone of inhibition [94, 97, 99]. The area around the disc, known as the inhibition zone, is considered the *δ* index, as shown in Figure 3.

### 4.3. Time-Kill Test

The time-kill kinetics assay is a dynamic method used to measure an agent's antimicrobial activity over time rather than just at one point in time as measured by static susceptibility tests; it measures bacterial viability over multiple time points and is important for determining whether an agent is bacteriostatic (a growth inhibitor) or bactericidal (a killing agent), optimizing dosage schedules, and evaluating the interactions between different antimicrobials for antagonistic or synergistic effects [100, 101].

**Figure 4:**
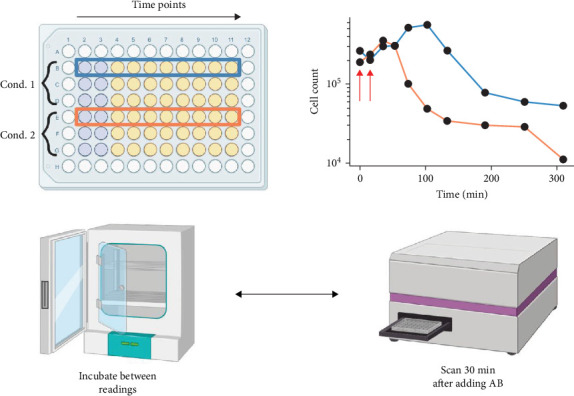
Overview of the time-kill assay procedure for evaluating antimicrobial activity, showing inoculation of bacterial cells in multiwell plates, addition of antimicrobial agents at specific time points, and measurement of bacterial viability over time. Data are used to generate bacterial count versus time profiles for each experimental condition. Adopted from [105].

Because it influences the choice of concentrations tested (usually MIC, 2× MIC, and 4× MIC) in the kinetic assay, the MIC of the antimicrobial agent must be determined before performing the time-kill assay [102–104]. The assay starts with the creation of a standardized bacterial suspension, which is usually diluted in nutrient broth and adjusted to a 0.5 McFarland standard, as shown in Figure 4. Then, using the previously established MIC values, antimicrobial agents are tested at fixed concentrations. After mixing the antimicrobial solution with the bacterial suspension, the mixture is incubated under carefully monitored conditions, typically between 28 and 37°C. To count viable CFUs, samples are collected at predefined intervals (e.g., 0, 2, 4, 6, 8, and 24 h), diluted if required, and then plated onto agar. Killing curves are created by plotting the results as the logarithm of CFU versus time. The percentage reduction in bacterial counts at each interval is calculated according to Equation (1) [106, 107].

(1) $$\begin{array}{c} \text{Percentage reduction}=\frac{\text{Initial count}-\text{count at time }t}{\text{Initial count}}\times 100. \end{array}$$

This assay provides valuable kinetic data, revealing how quickly and effectively an antimicrobial agent kills or inhibits bacteria. While it offers clinically relevant insights beyond single-time-point tests, it can be labor-intensive and does not replicate fluctuating drug concentrations in vivo or the complexities of the host environment. Additionally, it does not clarify the mechanisms of killing, which should be considered when interpreting the results [108].

### 4.4. Flow Cytometry

A quantitative analytical method called flow cytometry is used in conjunction with common antimicrobial assays like disc diffusion, dilution, and time-kill techniques. Size, granularity, fluorescence, and other cellular parameters can all be measured quickly and simultaneously with it, which makes it especially useful for evaluating single-cell metabolic activity, membrane integrity, and microbial viability [109, 110].

**Figure 5:**
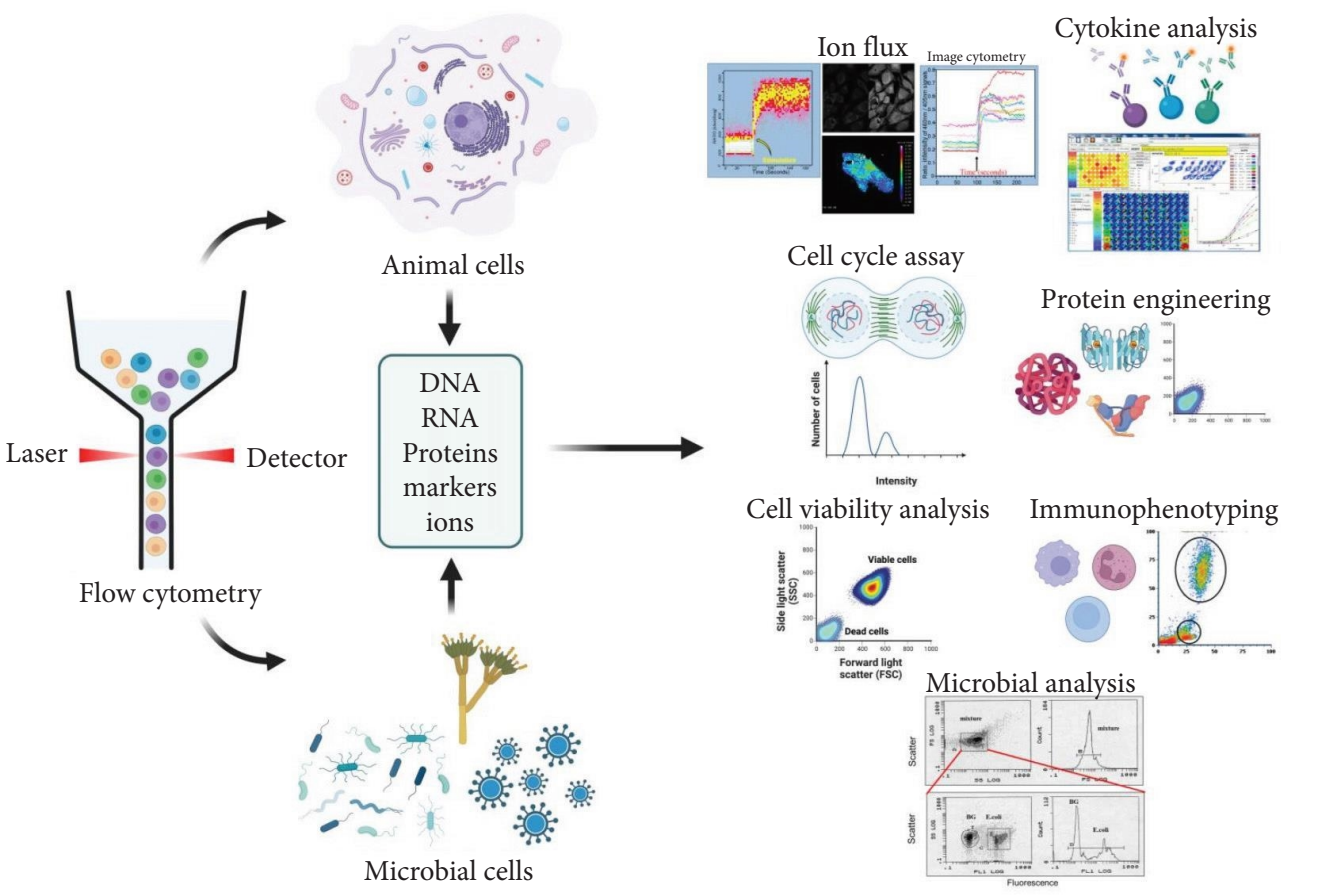
Broad applications of flow cytometry, including antibacterial testing. This technique is widely used in microbial viability assessment, cellular analysis, phenotyping, and various biological assays. Adopted from [111].

According to Figure 5, which illustrates the flow cytometry process, adopted from [111], a typical protocol involves exposing the target microorganism to the antimicrobial agent for a defined period, with untreated controls processed in parallel. After treatment, cells are stained with viability dyes such as SYTO 9, which penetrates intact membranes and produces green fluorescence, and propidium iodide (PI), which enters only cells with compromised membranes and emits red or orange fluorescence. The stained suspension is passed through the flow cytometer, where cells are hydrodynamically focused into a single-file stream and interrogated by one or more lasers. Emitted fluorescence and scattered light are detected by photomultiplier tubes or photodiodes, enabling discrimination between live and dead populations. Equations (2) and (3) describe the calculation of the proportions of viable and nonviable cells, respectively [112, 113]:

(2) $$\begin{array}{c} \text{Percentage of viable cell}=\frac{\text{Number of live cells}}{\text{Total number of cells}}\times 100, \end{array}$$

(3) $$\begin{array}{c} \text{Percentage of dead cells}=\frac{\text{Number of dead cell}}{\text{Total number of cells}}\times 100. \end{array}$$

The resulting data quantify the proportion of viable and nonviable cells, supporting antimicrobial efficacy evaluation and mechanistic studies. While the method offers high-throughput, precise, and reproducible single-cell analysis, it requires costly instrumentation, trained personnel, and careful calibration. Additionally, reagents may affect cell physiology, and biofilm studies require further optimization [111, 114, 115].

### 4.5. ATP Bioluminescence Assay

The ATP-bioluminescence assay is another sensitive quantitative method for determining antibacterial activity that utilizes an enzyme-catalyzed reaction (catalyzed by luciferase) that is highly dependent on the presence of ATP, a molecule present in all living cells [116]. In this technique, bacteria are genetically engineered to produce the luciferase enzyme either through the introduction of a gene or plasmid encoding for luciferase into their genome so that they continuously produce the enzyme. The assay involves the oxidation of luciferin by oxygen and magnesium ions in the presence of ATP, catalyzed by luciferase, to form oxyluciferin with yellow–green light emission [117].

**Figure 6:**
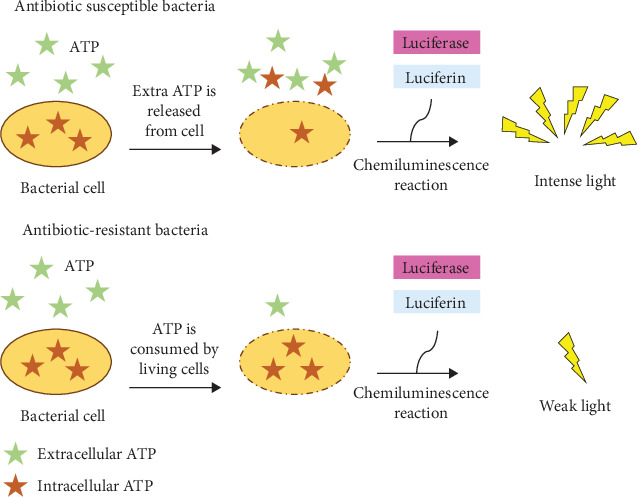
Principle of ATP-bioluminescence in antibiotic susceptibility testing, where light emission reflects ATP levels in susceptible versus resistant bacteria. Adopted from [118].

According to Figure 6, adopted from [118], the intensity of the emitted light is directly proportional to the ATP content of the sample, which reflects the metabolic activity and viability of the bacteria. Exposure to an antibacterial agent can kill some bacterial cells, thereby reducing ATP levels. Consequently, treated samples emit less light than untreated control samples. The bioluminescent signal is quantitatively measured using a luminometer or a bioluminescence imaging system, which records light emission over a defined time period [119].

Despite its sensitivity, the ATP-bioluminescence assay has several drawbacks. One major disadvantage is that bacterial strains must be prepared through genetic engineering. This process can be time-consuming and technically challenging, and it often requires customization for each bacterial species. Another limitation is signal interference. The phenomenon known as quenching occurs when compounds found in biological samples absorb or scatter light, lowering the intensity of the signal being measured. Additionally, some of the nonmicrobial components of the sample may glow on their own, leading to inaccurate results interpretation and erroneously high readings [120].

Numerous biological and technical factors may have an impact on this method. Depending on the bacteria's physiological condition, ATP levels can change. Because of its relative instability, the luciferase enzyme may have an impact on long-term experiment reproducibility. Enzyme activity can also be influenced by environmental variables like pH, temperature, and the strength of ions. Additionally, luminescence-enhancing or -inhibiting molecules may result in false-positive or false-negative test results. Consequently, accurate data interpretation requires careful experimental design, the application of suitable controls, and adequate validation [108].

### 4.6. B-TLC

Especially in the field of natural product research, B-TLC is a flexible and effective method for identifying and detecting antimicrobial compounds in complex mixtures. Because it enables simultaneous chromatographic separation and bioactivity assessment, it is particularly helpful for analyzing samples that contain several unknown components. This technique allows for the precise localization of biologically active molecules by combining a bioassay carried out directly on the same plate with thin-layer chromatographic separation of sample components. The antimicrobial potential of essential oils, plant-derived extracts, and bacterial secondary metabolites has been extensively screened using B-TLC [121, 122].

**Figure 7:**
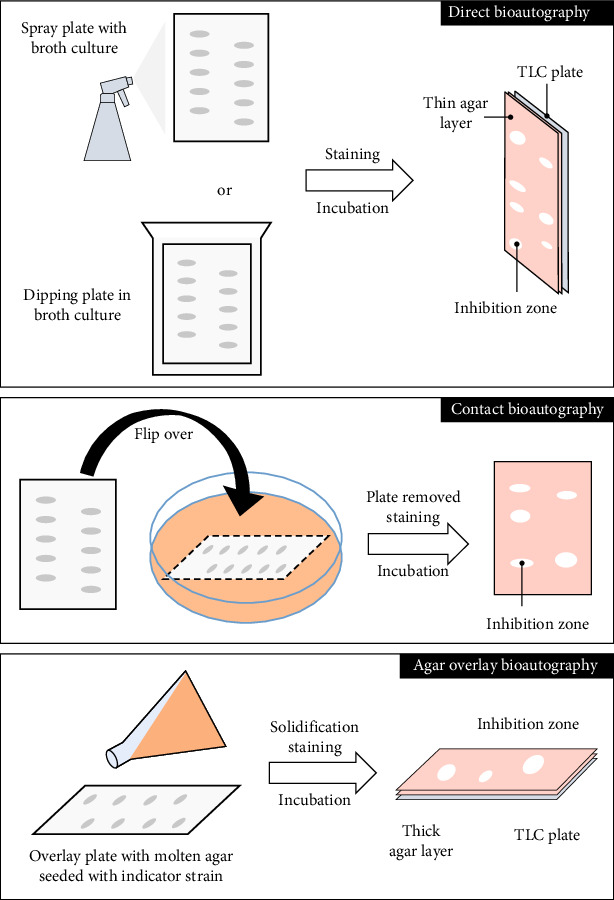
Overview of TLC-bioautography methods for antimicrobial detection, including direct spraying or dipping, agar diffusion contact, and immersion with molten agar. Active compounds are identified by zones of microbial growth inhibition. Adopted from [108].

According to Figure 7, which provides a schematic representation of the B-TLC process, adopted from [123], this procedure involves applying a small quantity of the sample to a TLC plate. The plate is then developed in an appropriate solvent system, leading to the separation of the constituents based on their polarity and interaction with the stationary phase. The resulting spots can be visualized under ultraviolet light or by using specific staining reagents. Each compound's migration is expressed as a retention factor (Rf), calculated as the ratio of the distance traveled by the compound to the distance traveled by the solvent front. While Rf values can differentiate components chemically, they do not indicate biological activity [124–126].

One of the three primary bioautographic techniques is applied to the produced TLC plate after separation. In the direct bioautography method, a suspension of the test microbe is either sprayed onto or submerged in the plate. After that, the plate is incubated for 18–24 h at a temperature of 35–37°C with high humidity. In accordance with their locations on the chromatogram, antimicrobial agents will show up as distinct zones of growth inhibition [123]. In contact bioautography, the plate is placed face down on an agar layer previously inoculated with the target microorganism. It is left in contact for 30–60 min to allow diffusion, after which it is removed. The agar is then incubated to reveal zones of inhibition [127, 128].

In immersion or overlay bioautography, the TLC plate is either immersed in or overlaid with molten agar that is seeded with the test microorganism. Visible inhibition zones are produced when active compounds diffuse into the agar following solidification and incubation [129]. When direct bioautography is not feasible, this final technique is especially helpful. This can happen when fungal mycelia obstruct spray nozzles or when reducing the risk of contamination is crucial. However, the dilution and dispersion of active compounds in the agar matrix may compromise the sensitivity of this method. For spore-forming fungi and pigmented bacteria, inhibition zones can be seen directly in all three methods, or they can be made visible with tetrazolium salts [130].

B-TLC has several advantages, such as identifying antimicrobial activity within complex matrices, rapid and sensitive detection, cost-effectiveness, applicability to a variety of sample types from natural or synthetic sources, and the ability to detect compounds at low concentrations; however, this technique is primarily qualitative, which does not quantify antimicrobial potency, so direct comparison between different compounds or determination of optimal concentration can be difficult, the sensitivity and reproducibility of the method are highly dependent on microbial growth conditions, and compounds that show no activity under those specific assay parameters will go undetected. Furthermore, sample matrix effects, extraction procedures, and the choice of TLC solvent system can all influence the results [108].

### 4.7. CFU

CFU indicates the number of individual colonies of each microorganism that develop on a media plate [131]. To determine the count of colony formation, the microbes must first grow on the culture media, and only viable cells are included in the final count. Images of the plate displaying the colonies can be captured and analyzed using colony counting software to obtain the total number of colonies [132].

## 5. Antibacterial and Antifungal Properties of Pure Zeolites

### 5.1. CZ

Narin et al. [133] examined the antibacterial properties of CZ, using particle sizes ranging from 75 to 150 μm, against *B. subtilis* and *E. coli* bacteria. Before conducting the antibacterial tests, the CZ particles were washed with hot deionized (DI) water and then dried over 24 h at 65°C. The diffusion technique was utilized to evaluate the antibacterial properties of CZ; however, the *δ* value was reported to be ~0 mm. As a result, it can be inferred that pure CZ exhibits minimal antibacterial activity.

### 5.2. ZA

In 2016, Alswat et al. [134] examined the antibacterial activity of ZA on *E. coli* (1), *S. choleraesuis* (1), *B. subtilis* (1), and *S. aureus* (1) using the diffusion method. Paper discs were immersed in suspensions containing ZA at a concentration of 10 mg/mL and allowed to dry for 24 h. The results indicated that ZA did not exhibit significant antibacterial efficacy against the tested bacteria.

In a separate study conducted in 2017, the antibacterial properties of ZA powder having an average particle size below 45 µm were evaluated using the diffusion method. The antimicrobial effects of ZA were tested against *B. subtilis* (1) and *S. choleraesuis* (1), using streptomycin (100 mg/mL) as the positive control and distilled water as the negative control. The results showed that ZA demonstrated minimal antimicrobial effects against the tested bacteria [135].

Another research study aimed to investigate the antibacterial properties of ZA powders on *S. aureus* (2) and *E. coli* (2) bacteria using the CFU method. For the antibacterial testing, a concentration of 0.1 mg/mL of zeolite was introduced to the bacterial suspension and incubated at 37°C for 1, 2, and 3 h. Compared to the control group, it was found that ZA did not demonstrate any significant antibacterial activity, as 100% of the bacteria remained viable [136].

Additionally, the antimicrobial properties of ZA were evaluated on oral bacteria in anaerobic environments using the dilution method. The bacterial strains tested included *P. intermedia*, *P. gingivalis*, *A. actinomycetemcomitans*, *S. mutans*, *A. viscosus*, *S. sanguis*, and *S. aureus*, which are considered major periodontal pathogens. The MIC for ZA was found to be 16,384 µg/mL for *P. intermedia* and *P. gingivalis* after 24 h for the other bacteria [137].

### 5.3. ZS

In a 2017 study, the antimicrobial properties of ZS on *E. coli*, *P. aeruginosa* bacteria, and *C. albicans* fungus were assessed. To conduct the evaluation, 1.5 g of zeolite was dispersed in 1000 mL of DI water. Subsequently, ZS samples were prepared in disc form (0.2 g/disc) using uniaxial compression for 30 s at 499 MPa with a manual hydraulic press. The results of the test indicated that ZS does not exhibit significant antibacterial efficacy against the tested microorganisms [138].

### 5.4. ZY

Salim and Malek conducted a study to investigate the antimicrobial activities of ZY with a Si/Al ratio of 2.9 against *E. coli* (3) and *S. aureus* (2) in saline solution and DI water using the dilution method. Initially, 5 g of ZY was added to 500 mL of 5 M sodium chloride (Cl) solution and shaken for 24 h at 25°C. Subsequently, it was diluted to concentrations ranging from 0.01 to 8 g/L in test tubes. The MIC values for ZY in both media were found to be higher than 12,000 μg/mL [139].

According to a recent study, Nik Malek et al. [140] assessed the antibacterial activity of ZY against *E. faecalis* (1), *P. aeruginosa* (1), *E. coli* (3), and *S. aureus* (2) using the diffusion method. The results demonstrated that ZY did not exhibit antibacterial activity, as no inhibition zone was observed.

In another article, the antimicrobial activity of zeolite ZY with a Si/Al ratio of 2.83 on *B. subtilis*, *E. coli*, *S. cerevisiae*, and *C. albicans* was examined. The test results revealed that pure ZY did not demonstrate significant antimicrobial properties [45].

### 5.5. ZX

An article aimed to illustrate the antimicrobial activity of ZX against bacteria *B. subtilis*, *E. coli*, and yeast *S. cerevisiae* and *C. albicans*. The antimicrobial properties of ZX with a Si/Al ratio of 1.64 were evaluated using the dilution method. The results indicate that ZX does not exhibit significant antimicrobial activity [45].

Table 3 summarizes the antibacterial properties of various pure zeolites, categorized by zeolite properties, type of microorganism, zeolite concentration, and reported results from previous studies.

## 6. Antibacterial and Antifungal Properties of Ion-Exchanged Zeolites

Ion-exchanged zeolites demonstrate strong antibacterial properties, unlike pure zeolites, which show little to no antimicrobial activity. Various metallic cations, such as Ag^+^, Zn^2+^, Cu^2+^, and Ni^2+^, have been extensively studied for their effectiveness in this area. There are significant differences in cation exchange capacity (CEC) and the release profiles of these ions between natural and synthetic zeolites, both of which critically impact their antimicrobial performance.

To improve ion-exchange efficiency, both submicron and nanoparticle forms of metal ions have been employed across various types of zeolites. Many heavy metal ions exhibit natural antibacterial and antifungal properties. When these ions are incorporated into the porous structure of zeolites, they create a controlled and sustained release mechanism [31, 141]. This approach not only enhances antimicrobial activity but also highlights the potential use of zeolites in biomedical applications. The antimicrobial effects mainly involve the disruption of microbial cell membranes and the inhibition of important enzymatic functions [142, 143]; in the case of fungi, the interference with ergosterol biosynthesis, which is a vital component of the fungal cell membrane [144, 145]. Consequently, most cutting-edge researches on ion-exchanged zeolites have concentrated on the types of metallic cations incorporated, as these are key factors that determine their overall antimicrobial effectiveness.

### 6.1. Ag–Zeolite

In a separate investigation, the in vitro antibacterial properties of Ag–ZA against methicillin-resistant *S. aureus* were assessed. Titanium alloy (TA) discs, measuring 8 mm in diameter and 1 mm in thickness, were prepared and coated with Ag–ZA. These discs were rinsed with DI water for ~15 min and then dried using air compression. The zeolite-coated TA (ZA–TA) discs were subsequently immersed in a 0.001 M AgNO_3_ solution, referred to as Ag–ZA–TA. Three types of discs were used: TA, ZA–TA discs, and Ag–ZA–TA. At the conclusion of the experiments on Day 6, it was observed that about 72.8% of the Ag ion content loaded onto the zeolite, with a weight percentage of 2.3%, had been released. After 4 h of incubation, the live bacteria concentration in the suspension with Ag–ZA–TA was markedly lower compared to the other two groups, demonstrating effective inhibition of bacterial growth [146].

Another study assessed the antibacterial activity of ZA containing 2.5 wt.% Ag ions against various bacteria, including *P. intermedia*, *P. gingivalis*, *A. actinomycetemcomitans*, *S. mutans*, *A. viscosus*, *S. sanguis*, and *S. aureus*, by determining their MICs. The MIC values were measured after 48 or 72 h for *A. actinomycetemcomitans*, *P. intermedia*, and *P. gingivalis*, and after 24 h for the remaining bacteria. The findings revealed that the MIC values for *P. intermedia*, *P. gingivalis*, and *A. actinomycetemcomitans* were significantly lower compared to those of the other bacteria [137].

In 2016, Salim and Malek conducted research on the antibacterial properties of Ag–ZY against *S. aureus* (2) and *E. coli* (3) bacteria. They incorporated varying amounts of Ag ions into ZY with a Si/Al ratio of 10.2 and examined its antibacterial effects using the MIC method. Ag is widely recognized as a nontoxic inorganic antibacterial agent capable of combating ~650 types of pathogenic microorganisms [46, 147, 148]. Its wide range of activity against bacteria and fungi has driven its increasing application in medical fields, such as wound dressings and the prevention of bacterial colonization on medical devices. In this context, Ag-zeolite is regarded as one of the most efficient materials for removing bacteria from the environment [138–140, 147–149].

In a subsequent study, the antibacterial properties of Ag zeolite (Ag–ZA) and Ag nanoparticle zeolite (AgNP-ZA) against *S. aureus* (2) and *E. coli* (2) were evaluated. To synthesize Ag–ZA and AgNP–ZA, ion-exchange solutions containing 25, 50, 100, and 200 mg/L of AgNO₃ were prepared. Zeolite powder was dispersed in the AgNO₃ solutions at a concentration of 1% (w/v) and stirred continuously for 2 h at 25°C. For the synthesis of AgNP–ZA composites, prepared Ag–ZA was suspended in 100 μL of 2 M sodium borohydride, with Ag^+^ ions added gradually under vigorous stirring for 24 h at 25°C to achieve in situ size reduction. A bacterial suspension was introduced to the compounds at a concentration of 0.1 mg/mL for 1, 2, and 3 h at 37°C, and the number of viable bacteria was measured using the CFU method. The results indicated that, for *E. coli*, all composites except Ag–ZA at a concentration of 25 mg/L resulted in over 80% mortality after 1 h of contact time. Additionally, the highest antibacterial activity was observed with Ag–ZA at 200 mg/L AgNO_3_, which recorded 84% mortality after 3 h. In contrast, for AgNP–ZA, all concentrations ranging from 50 to 200 mg/L AgNO_3_ achieved 100% mortality after 3 h of exposure [136].

Demirci et al. investigated the antimicrobial properties of ZA with different Si/Al ratios after ion-exchanging it with Ag ions. They tested the efficacy against various microorganisms, including bacteria (*B. subtilis*, *S. aureus*, *E. coli*, and *P. aeruginosa*), yeast (*C. albicans*, *C. glabrata*), and fungi (*P. vinaceum*, *A. niger*) using the MIC method. For their study, a 1 M solution of Ag nitrate (AgNO_3_) was prepared for the ion-exchange process. Two variants of ZA, designated as ZA1 and ZA2, were synthesized, with Si/Al ratios of 0.84 and 1.6, respectively. To test antimicrobial activity, microbial media were prepared by suspending 2048 μg/mL of Ag–ZA in 10 mL of tryptic soy broth (TSB) for bacteria and in 10 mL of Sabouraud dextrose broth (SDB) for fungi. The reported MIC values for the microorganisms tested ranged from 16 to 512 μg/mL, with the lowest MIC of 16 μg/mL observed for *Bacillus subtilis* when using Ag–ZA1 [150].

The preparation of different types of Ag–ZY was conducted by adding 1 g of zeolite to 100 mL of AgNO_3_ solutions with varying initial concentrations of AgNO_3_ (Ag(1): 100 mg/L, Ag(2): 600 mg/L, and Ag(3): 900 mg/L). The MIC values for Ag(1)–ZY, Ag(2)–ZY, and Ag(3)–ZY against *E. coli* (3) were found to be 400, 50, and 10 μg/mL in DI water and 4000, 800, and 800 μg/mL in saline solution, respectively. For *S. aureus* (2), the MIC values of Ag(1)–ZY, Ag(2)–ZY, and Ag(3)–ZY were 4000, 400, and 50 μg/mL in DI water and 6000, 2000, and 2000 μg/mL in saline solution, respectively. The results indicate that Ag–ZY is more effective against *E. coli* (3) than *S. aureus* (2) in DI water. However, in the saline solution, Ag–ZY showed no antibacterial activity against either of the bacteria due to the presence of Cl ions in the solution [139].

A study conducted by Ferreira et al. [45] aimed to assess the antibacterial potential of Ag–ZA against the bacteria *B. subtilis* and *E. coli*, as well as the yeasts *S. cerevisiae* and *C. albicans*, using the agar dilution method. To prepare Ag–ZY, 2.5 g of ZY (with a Si/Al ratio of 2.83) were mixed with 50 mL of a 0.05 M AgNO_3_ solution (20 mL of solution for each gram of zeolite). Various concentrations of Ag–ZY, ranging from 0.1 to 1 mg/mL, were then incorporated into either lysogeny broth with agar or yeast extract peptone agar (YPDA) media, depending on the type of microorganism being tested. The results indicated that at a concentration of 0.2 mg/mL, all bacterial cells were killed in the Ag–ZY samples, while the MIC value for both yeasts was 100 μg/mL. Thus, the synthesized Ag–ZY demonstrated significant antimicrobial activity against the tested microorganisms, outperforming similar studies that utilized other types of zeolites.

Demirci et al. [150] conducted a study to evaluate the antimicrobial properties of ZX that had been ion-exchanged with Ag ions (Ag–ZX). This study tested its effectiveness against a variety of microorganisms, including bacteria (*B. subtilis*, *S. aureus*, *E. coli*, and *P. aeruginosa*), yeast (*C. albicans*, *C. glabrata*), and fungi (*P. vinaceum*, *A. niger*). Two variants, ZX1 and ZX2, were synthesized with Si/Al ratios of 3.2 and 8, respectively. The results revealed that the MIC values for the tested microorganisms ranged from 16 to 1024 µg/mL. The lowest MIC was found to be 16 µg/mL for *P. aeruginosa* when treated with Ag-ZX1. Furthermore, ZX1 was more effective than ZX2 against most of the tested microorganisms.

Ferreira et al. [45] investigated the antibacterial properties of Ag–ZX against the bacteria *B. subtilis* and *E. coli*, as well as the yeasts *C. albicans* and *S. cerevisiae*. To prepare Ag–ZX, 2.5 g of ZX (with a Si/Al ratio of 1.64) were mixed with 50 mL of a 0.05 M AgNO_3_ solution. The MIC was reported to be 1000 μg/mL for both yeasts. Additionally, no viable bacterial cells were observed for either of the bacteria at a concentration of 300 μg/mL of Ag–ZX.

A recent study investigated the antibacterial activity of Ag–ZS, which has a high Si/Al ratio, against the bacteria *P. aeruginosa* and *E. coli*, as well as the fungus *C. albicans*. The ion-exchange process involved adding 5 g of zeolite to 1 liter of a 0.1 N AgNO_3_ aqueous solution. This mixture was then kept at 70°C for 6 h, followed by magnetic stirring in the dark. The results demonstrated a significant inhibition zone of ~1 cm in diameter around the disc after 24 h for *P. aeruginosa* and a 0.5 cm diameter zone for *E. coli*. Additionally, the inhibition zone for the fungus *C. albicans* was about 0.3 cm in diameter around the disc [138].

Another recent article investigated the antibacterial properties of EZ with Ag ion (Ag–EZ). The ion-exchange procedure involved adding 20 mL of 0.05 M Ag perchlorate solutions to various EZ suspensions. After specific durations and treatments, the study demonstrated that no viable cells were found at certain incubation times for ion-exchanged samples [151].

Copcia et al. [34] investigated the antimicrobial effects of Ag-modified natural CZ (Ag–CZ) against *E. coli* and methicillin-resistant *S. aureus* cells. They began by modifying zeolite powder with a particle size of 0.1 mm using a 1 M solution of oxalic acid (H_2_C_2_O_4_·2H_2_O), referred to as P1. This was followed by curing with a 1 M sodium hydroxide (NaOH) solution, denoted as P2, at 100°C for 5 h, maintaining a liquid-to-solid ratio of 5:1 to enhance the CEC of the zeolite. Subsequently, they suspended the modified zeolite samples in a 0.1 M Ag nitrate (AgNO_3_) solution for 24 h at 25°C in the dark, shaking them intermittently to produce Ag-loaded zeolite samples, P1-Ag^+^ and P2-Ag^+^. The findings indicated that a concentration of 2 mg/mL of Ag-CZ effectively inhibited the growth of both *E. coli* and *S. aureus* in the tested media. Additionally, both P1-Ag^+^ and P2-Ag^+^ samples exhibited significant antibacterial effects against the two bacteria. Specifically, at a concentration of 0.1 mg/mL, the P1-Ag^+^ sample resulted in a mortality rate of 67.42% CFU/mL for *E. coli* and 82% CFU/mL for *S. aureus*. In contrast, the same concentration of the P2-Ag^+^ sample showed mortality rates of 37.29% CFU/mL for both *E. coli* and *S. aureus*.

Table 4 summarizes the antibacterial efficacy of Ag–zeolites, categorized by zeolite characteristics, microorganism types, zeolite concentration, and literature-reported findings.

### 6.2. Zn–Zeolite

A 2019 study investigated the antimicrobial effects of Zn ion-exchanged ZX (Zn–ZX) against the bacteria *E. coli* and *S. aureus*. To prepare Zn–ZX, 2 g of ZX were mixed with a 0.15 mol/L solution of ZnSO_4_·7H_2_O (W solution/W zeolite = 25) at a temperature of 70°C. After 1 h, the Zn–ZX sample was obtained through a process of filtering, rinsing, and drying. Different amounts of the Zn–ZX samples were then added to bacterial solutions, resulting in Zn ion concentrations ranging from 10 to 1000 ppm for *E. coli*. The results showed that with 500 ppm of Zn–ZX, the bacterial growth in *E. coli* decreased from 7.5 log to 4.75. Similarly, for *S. aureus*, at a concentration of 1000 ppm of Zn–ZX, bacterial growth also decreased from 7.5 log to 4.75 [152].

Demirci et al. [150] evaluated the antimicrobial properties of ZX with different Si/Al ratios that were ion-exchanged with Zn ions. They tested the ZX against various microorganisms, including bacteria (*B. subtilis*, *S. aureus*, *E. coli*, and *P. aeruginosa*), yeast (*C. albicans*, *C. glabrata*), and fungi (*P. vinaceum*, *A. niger*). For the ion-exchange reaction, they prepared a 1 M ZnCl_2_ solution and synthesized two variants, ZX1 and ZX2, with Si/Al ratios of 3.2 and 8, respectively. The MIC values for the tested microorganisms ranged from 1024 to 2048 μg/mL.

Hrenovic et al. [153] examined the antimicrobial properties of natural zeolite tuff, which contains 70 wt.% clinoptilolite with a particle size ranging from 0.063 to 0.1 mm. The zeolite was modified with Zn ions (Zn–CZ) to enhance its effectiveness against *S. aureus* and *E. coli*. To prepare Zn–CZ, 1 g of zeolite was added to 100 mL of a 6 mM ZnCl_2_ aqueous solution and shaken at 30°C for 24 h. To improve the ion-exchange capacity of clinoptilolite tuff (CZ), it was further converted into a sodium-rich zeolite. In the experimental setup, 1 mL of suspended biomass of *E. coli* or *S. aureus* was inoculated into 100 mL of various liquid media, including synthetic wastewater, effluent water, and Luria–Bertani medium, with the initial concentration of CFUs ranging from 10^6^ to 10^7^ mL^−1^ in Schott bottles. After 1 h of contact with Zn–CZ, a significant reduction in the number of viable *E. coli* and *S. aureus* cells was observed. However, the number of *S. aureus* cells in the effluent water and synthetic wastewater did not show a significant difference after either 1 or 24 h of contact. The study demonstrated that the effluent water exhibited the greatest reduction in bacterial count, followed by synthetic wastewater, with Luria–Bertani medium showing the least reduction. Therefore, it can be concluded that Zn–CZ has effective antibacterial activity against the tested bacteria and holds potential as a disinfectant for secondary effluent water.

Table 5 illustrates the antibacterial effects of Zn–zeolites with respect to their physicochemical properties, tested microorganisms, applied concentrations, and findings from prior studies.

### 6.3. Cu–Zeolite

The antimicrobial effectiveness of Cu ion-modified ZX (Cu–ZX) was evaluated against the bacteria *S. aureus* and *E. coli* using a specified method. This process involved dispersing 2 g of ZX in a 0.2 mol/L CuCl_2_·2H_2_O solution (with a liquid-to-solid ratio of 20) to produce Cu–ZX, carried out at 70°C. The results demonstrated that when 1000 ppm of Cu–ZX was applied, the logarithmic growth of *E. coli* decreased from 7.5 to 3.5 after 1 h of contact. For *S. aureus*, the application of 100 ppm of Cu–ZX resulted in a reduction of bacterial logarithmic growth from 7.5 to 2 after 1 h. Furthermore, Demirci et al. [150] assessed the antimicrobial properties of ZX1 and ZX2, which have Si/Al ratios of 3.2 and 8, respectively, after they were ion-exchanged with Cu ions (Cu–ZX) and tested against various microorganisms. The MIC values for the microorganisms tested ranged from 256 to 1024 μg/mL.

Hrenovic et al. [153] investigated the antibacterial properties of natural zeolite modified with Cu ions (Cu–CZ) against *S. aureus* and *E. coli* bacteria. To create the metal-loaded zeolites, they added 1 g of sodium-rich zeolite to 100 mL of a 6 mM CuCl_2_ aqueous solution. The mixture was then shaken at 30°C for 24 h. The study found that the number of viable *S. aureus* and *E. coli* cells decreased after 1 h of contact with Cu–CZ. Additionally, the reduction in bacterial count was more pronounced in effluent water and synthetic wastewater compared to LB medium. The decline in *S. aureus* cell numbers did not show significant differences between effluent water and synthetic wastewater after both 1 and 24-h contact periods. The study concluded that Cu–CZ could be effectively utilized for disinfecting secondary effluent water.

Demirci et al. [150] assessed the antimicrobial properties of ZA1 and ZA2, which had Si/Al ratios of 0.84 and 1.6, respectively, after ion-exchanging with Cu ions. The types of microorganisms used and the testing process were conducted in the same manner as previously described. The results indicated that the MIC values for the tested microorganisms ranged from 256 to 1024 μg/mL.

In a recent study, the antibacterial properties of Cu–ZY with different amounts of Cu ion were investigated for *E. coli* (3), *P. aeruginosa* (1), *S. aureus* (2), and *E. faecalis* (1) bacteria based on the disc diffusion technique. Initially, ~3.798 g of Cu(NO3)_2_ was dissolved in 900 mL of DI water. Different concentrations of Cu–ZY were prepared by using 100, 600, and 900 ppm of Cu for the ion-exchange process. The results showed the inhibition zones for *E. coli* (3) at 1.60 mm, 1.55 mm, and 1.45 mm for the 100, 600, and 900 ppm concentrations, respectively. For *P. aeruginosa* (1), the inhibition zones were 2.15 mm, 2.00 mm, and 1.90 mm, and for *S. aureus* (2), they measured 2.40 mm, 2.25 mm, and 2.20 mm. Lastly, for *E. faecalis* (1), the inhibition zones were recorded as 1.50 mm, 1.95 mm, and 2.25 mm, corresponding to the same concentrations [140].

Table 6 details the antibacterial properties of Cu–zeolites, classified by zeolite characteristics, types of microorganisms, concentrations used, and results reported in the literature.

### 6.4. Nickel (Ni)–Zeolite

The antimicrobial properties of Ni-loaded zeolites (Ni–zeolites) have been studied to a limited extent. However, a study conducted by Hrenovic et al. [153] investigated the antibacterial effects of natural zeolite modified with Ni ions (Ni–CZ) against *S. aureus* and *E. coli* bacteria. To prepare the metal-loaded zeolites, 1 g of zeolite was mixed with 0.1 L of a 6 mM NiCl_2_ aqueous solution and stirred at 30°C for 24 h. The antimicrobial activity of Ni–CZ was evaluated in three different liquid media, namely Luria–Bertani (LB) medium, synthetic wastewater, and effluent water. The results indicated that after 1 h of contact with the bacteria, only a negligible reduction in bacterial counts was observed across all media, with the lowest activity detected in the LB medium. After 24 h of exposure, the antibacterial effect increased slightly; however, the results showed that the reduction in bacterial numbers remained below 20% in all cases. These findings suggest that Ni–CZ demonstrates limited antibacterial efficacy in liquid environments, particularly under nutrient-rich conditions such as those found in LB medium, and may therefore be ineffective as a disinfectant for effluent water treatment. This observation is further supported by the data obtained from effluent water after 24 h.

Table 7 summarizes the antibacterial activity of a Ni–zeolite based on the single available study, including zeolite features, microorganism types, concentration, and reported results.

## 7. Results and Discussion

In the literature survey that we have performed in this study, the antibacterial activities of ion-exchanged zeolites are compared together in order to find out the superiority and inferiority of various cations exchanged in different types of zeolites. As a result of our investigation, we try to specify the most critical factors affecting the antibacterial efficacy of ion-exchanged zeolites. These factors are those that basically define the CEC of zeolites.

Several aforementioned studies have investigated the impact of the Si/Al ratio on the antibacterial properties of ion-exchanged zeolites. Generally, a lower Si/Al ratio enhances the CEC of zeolites. Consequently, ion-exchanged zeolites with a lower Si/Al ratio often exhibit superior antibacterial properties compared to those with higher ratios. This improvement is attributed to the negative charge on the aluminum structure, which facilitates greater ion exchange with the zeolite, thereby increasing antibacterial activity. This result is corroborated by the data presented in Tables 4–7. Since the Si/Al ratio of both synthetic and natural zeolites is partially changeable through various chemical modifications, for example, acid modification, we could basically tailor the CEC and eventually the antibacterial efficacy of every individual zeolite. This property provides zeolites a superior priority to many other materials utilized as antibacterial agents.

By comparing Tables 4–7, we can conclude that the type of ion is also a significant factor influencing the antimicrobial properties of ion-exchanged zeolites. Among the metallic ions examined in this study, it appears that Ag is the most effective antibacterial agent, followed by Zn, Cu, and Ni. Noticeably, ion nanoparticles demonstrate limited antimicrobial properties compared to standard-sized ions (as can be seen in Table 4). This observation may be attributed to the greater ability of normal-sized metallic cations to disrupt the cell walls of microorganisms compared to ion nanoparticles.

As another result of our literature survey, we can claim that ion-exchanged zeolites exhibit more effective antibacterial properties than pure metallic ions. This phenomenon is clearly presented in the study by Wang et al. [146], who investigated the antibacterial effects of pure Ag and Ag–zeolite ions individually. Their results indicate that Ag–zeolite has superior antibacterial properties. This phenomenon could be addressed by the sustained release of Ag ions from Ag–zeolite, which enhances the antimicrobial activity compared to pure Ag ions eventually [146].

In addition to the abovementioned factors, we found that the solution medium used in the cation exchange procedure can significantly affect the antibacterial properties of ion-exchanged zeolites. This fact is well demonstrated by Salim and Malek [139], who investigated the difference between DI water and a saline solution (0.9%) to evaluate the antimicrobial activity of Ag zeolite. Their findings revealed that Ag-zeolite performs more effectively in DI water. In saline solution, negatively charged Cl ions are present, which attract positively charged Ag ions (Ag^+^). As a result, the availability of Ag ions to penetrate and attack bacterial cell walls is reduced in saline solution. As a result of our literature survey, this issue is almost neglected by researchers trying to develop ion-exchanged zeolites with high antibacterial properties. In another word, researchers could try novel cation exchange solutions to enhance the loaded cations on their zeolites and thereby enhance their antibacterial properties.

Cation loading (CL) plays a vital role in improving the bioavailability of metallic ions in microbial solutions. Several operational conditions directly influence the CL of ion-exchanged zeolites, such as temperature, concentration of the ion solution, and the duration of the ion exchange process. However, these parameters become ineffective if the zeolite molecules reach full saturation with metallic cations. Our literature review reveals that none of these parameters has yet been explored in this context.

In addition to the previously mentioned factors, pretreatment of zeolites significantly affects their CEC by altering the crystalline framework. Copcia et al. [34] reported that Ag-exchanged clinoptilolite pretreated with oxalic acid demonstrated stronger antibacterial activity against both *E. coli* and *S. aureus* compared to the NaOH-pretreated sample. This effect was attributed to the fact that sodium ions have a limited ability to displace framework cations such as K^+^, Ca^2+^, Mg^2+^, and Fe^3+^, whereas oxalic acid effectively removes these ions, thereby increasing the available sites for Ag exchange. Consequently, the oxalic acid–pretreated zeolite incorporated more Ag, which enhanced its antibacterial performance against both Gram-negative and Gram-positive bacteria.

Although pH is a key parameter that can influence the antimicrobial activity of metal-loaded zeolites, particularly through mechanisms such as increased ion release and membrane disruption, it is often underreported. In the study by Hrenović et al. [153], final pH values were measured for each experimental condition in both *E. coli* and *S. aureus* assays. The values cited here refer specifically to the *E. coli* experiments. After 24 h of exposure in effluent water, the final pH values were 7.14 for Cu-loaded zeolite (CuNZ), 7.18 for Zn-loaded zeolite (ZnNZ), and 7.97 for Ni-loaded zeolite (NiNZ). While pH changes in some media ranged from 0.19 to 1.59 units compared to controls, the pH increase in effluent water was notably higher for the NiNZ group. However, the reduction in bacterial counts was not directly correlated with these pH variations. For example, NiNZ caused the largest pH increase but showed the weakest antibacterial activity compared to other groups, such as CuNZ and ZnNZ. These findings suggest that the type and inherent toxicity of the released metal ions play a more decisive role in bacterial inactivation than pH variation alone. Therefore, future studies should include detailed pH measurements to clarify the relative contributions of pH shifts, ion toxicity, and other possible mechanisms in the antimicrobial performance of ion-exchanged zeolites.

Moreover, our literature survey shows that some other factors have been missed in relevant publications, specifically factors that affect the rate at which ions are released from ion-exchanged zeolites. Alongside the concentration of ions loaded onto the zeolite, which is determined by the zeolite's CEC, the driving force created by the concentration gradient of cations in the surrounding solution is important too. This driving force depends basically on the content of the release milieu. Furthermore, it should be taken into consideration that the soluble ions and proteins present in the environment can deactivate the cations released from the ion-exchanged zeolites; and therefore, suppress the antimicrobial property of the released ions [137, 154, 155].

Table 8 provides a summary of the key factors that influence the antimicrobial and antibacterial properties of ion-exchanged zeolites.

Apart from the factors outlined in Table 8, it appears that the particle size of the zeolite may impact the CEC of the zeolite. However, this aspect has not yet been explored in previously published articles.

## 8. Conclusions

Ion-exchanged zeolites can be effective antibacterial agents for medical applications. In this process, an alkaline or alkaline earth cation is replaced with a metallic cation, which then interacts with the bacterial environment by releasing the metallic cation. One of the main advantages of using ion-exchanged zeolites, compared to other antibacterial materials, is their ability to sustain the release of the metallic cation, resulting in long-term effectiveness. Our study demonstrates straightforwardly that the synthetic ion-exchanged zeolite exhibits superior antibacterial properties compared to natural zeolite. Additionally, based on our results in this study, we can claim that pure synthetic as well as natural zeolite has a very limited antimicrobial properties compared with ion-exchanged ones. Nevertheless, some already published studies observed antibacterial of specifically pure natural zeolites. We are confident that this effect is seen due to the already loaded metallic cations on zeolites in natural mines, which may happen due to the thousands of years of environmental changes.

The results of our literature survey demonstrate that ion-exchanged zeolites can control the release of charged metallic cations through various structural and environmental factors, such as by modifying the Si/Al ratio of the zeolite. Nevertheless, some other important factors, for example cation exchange solution, temperature, as well as release medium, have been widely neglected in previous research on the antibacterial properties of zeolites.

Among the publications reviewed, it can be concluded that the highest antibacterial activity was observed in ZA and ZX, which were exchanged with Ag ions. These zeolites showed effectiveness against *Escherichia coli* and *Bacillus cereus* bacteria, with a MIC of ~16 µg/mL, outperforming other ion-exchanged zeolites.

## Figures and Tables

**Table 1:** Physical characteristics of zeolites.

Zeolite type	Name	Abbreviation	Framework	Chemical formula	Si/Al	Supercage (Å)
Natural	Clinoptilolite zeolite	CZ	Heulandite-type (HEU) [39, 59]	(Na, K)_6_Al_6_Si_30_O_72_·20H_2_O [60, 61]	2.7–5.3 [62]	4.5–6 [39]

Synthetic	Zeolite A	ZA	Linde type A (LTA) [9]	Na_12_(H_2_O)_27_|_8_[Al_12_Si_12_O_48_]_8_ [47]	≤2 [39]	3.5–4.5 [24]
	ZSM-5	ZS	ZSM-5-type (MFI) [28]	Na_*n*_Al_*n*_Si_96–*n*_O_192_·16H_2_O (0 < *n* < 27) [41]	1–100 [39]	4.5–6.0 [41]
	Zeolite Y	ZY	Faujasite (FAU) [28]	|(Mg, Ca, Na_2_)_29_(H_2_O)_240_|[Al_58_ Si_134_ O_384_] [41]	1.5–3.8 [45]	6–8 [41]
	Faujasite zeolite	FZ		(Na_2_, Ca, Mg)_3.5_[Al_7_Si_17_O_48_]·32(H_2_O) [41]	2.1–2.8 [28]	7.4 [41]
	Zeolite X	ZX		(Mg, Ca, Na_2_)_29_ (H_2_O)_240_| [Al_58_Si_134_O_384_] [41]	1–3 [63]	6–8 [41]
	EMT zeolite	EZ	Hexagonal Faujasite (EMT-type) [28]	|Na_20_(*C*_12_H_24_O_6_)_4_(H_2_O)_22.6_| [Si_76_Al_20_O_192_] [41]	2–5 [41]	7.5 [41]
	Zeolite beta	ZB	Beta-type (BEA) [28]	[xNa.(1x)]AlO_2_.ySiO_2_.wH_2_O [41] *X*≤1, 5<*y* < 100, *w* ≤ 4	5–100 [41]	6.7 [41]

**Table 2:** Abbreviation of microorganisms.

Name	Abbreviation	Classification
*Escherichia coli*	*E. coli*	*Bacteria* (*Gram-negative*)
*Escherichia coli* (E266)	*E. coli* (1)	*Bacteria* (*Gram-negative*)
*Escherichia coli* (ATCC 25922)	*E. coli* (2)	*Bacteria* (*Gram-negative*)
*Escherichia coli* (ATCC 11229)	*E. coli* (3)	*Bacteria* (*Gram-negative*)
*Enterococcus faecalis* (ATCC 29212)	*E. faecalis* (1)	*Bacteria* (*Gram-positive*)
*Staphylococcus aureus*	*S. aureus*	*Bacteria* (*Gram-positive*)
*Staphylococcus aureus* (S276)	*S. aureus* (1)	*Bacteria* (*Gram-positive*)
*Staphylococcus aureus* (ATCC 6538)	*S. aureus* (2)	*Bacteria* (*Gram-positive*)
*Staphylococcus aureus* (RN450)	*S. aureus* (3)	*Bacteria* (*Gram-positive*)
*Saccharomyces cerevisiae*	*S. cerevisiae*	*Fungus* (*Yeast*)
*Streptococcus sanguis*	*S. sanguis*	*Bacteria* (*Gram-positive*)
*Streptococcus sanguis* (ATCC 10556)	*S. sanguis* (1)	*Bacteria* (*Gram-positive*)
*Streptococcus mutans*	*S. mutans*	*Bacteria* (*Gram-positive*)
*Streptococcus mutans* (NCTC 10449)	*S. mutans* (1)	*Bacteria* (*Gram-positive*)
*Salmonella choleraesuis* (ATCC 10708)	*S. choleraesuis* (1)	*Bacteria* (*Gram-negative*)
*Bacillus subtilis*	*B. subtilis*	*Bacteria* (*Gram-positive*)
*Bacillus subtilis* (B29)	*B. subtilis* (1)	*Bacteria* (*Gram-positive*)
*Bacillus cereus*	*B. cereus*	*Bacteria* (*Gram-positive*)
*Pseudomonas aeruginosa*	*P. aeruginosa*	*Bacteria* (*Gram-negative*)
*Pseudomonas aeruginosa* (ATCC 15442)	*P. aeruginosa* (1)	*Bacteria* (*Gram-negative*)
*Porphyromonas gingivalis*	*P. gingivalis*	*Bacteria* (*Gram-negative*)
*Porphyromonas gingivalis* (GAI 7802)	*P. gingivalis* (1)	*Bacteria* (*Gram-negative*)
*Porphyromonas gingivalis* (381)	*P. gingivalis* (2)	*Bacteria* (*Gram-negative*)
*Porphyromonas gingivalis* (1992)	*P. gingivalis* (3)	*Bacteria* (*Gram-negative*)
*Porphyromonas gingivalis* (W-50)	*P. gingivalis* (4)	*Bacteria* (*Gram-negative*)
*Prevotella intermedia*	*P. intermedia*	*Bacteria* (*Gram-negative*)
*Prevotella intermedia* (ATCC 25611)	*P. intermedia* (1)	*Bacteria* (*Gram-negative*)
*Penicillium vinaceum*	*P. vinaceum*	*Fungus* (*mold*)
*Actinobacillus actinomycetemcomitans*	*A. actinomycetemcomitans*	*Bacteria* (*Gram-negative*)
*Actinobacillus actinomycetemcomitans* (ATCC 29522)	*A. actinomycetemcomitans* (1)	*Bacteria* (*Gram-negative*)
*Actinobacillus actinomycetemcomitans* (ATCC 29524)	*A. actinomycetemcomitans* (2)	*Bacteria* (*Gram-negative*)
*Actinobacillus actinomycetemcomitans* (NCTC 9710)	*A. actinomycetemcomitans* (3)	*Bacteria* (*Gram-negative*)
*Actinobacillus actinomycetemcomitans* (Y4)	*A. actinomycetemcomitans* (4)	*Bacteria* (*Gram-negative*)
*Actinobacillus actinomycetemcomitans* (99)	*A. actinomycetemcomitans* (5)	*Bacteria* (*Gram-negative*)
*Actinomyces viscosus*	*A. viscosus*	*Bacteria* (*Gram-positive*)
*Actinomyces viscosus* (IFM 1927)	*A. viscosus* (1)	*Bacteria* (*Gram-positive*)
*Aspergillus niger*	*A. niger*	*Fungus* (*mold*)
*Candida albicans*	*C. albicans*	*Fungus* (*yeast*)
*Candida glabrata*	*C. glabrata*	*Fungus* (*yeast*)
*Porphyromonas gingivalis* (381)	*P. gingivalis* (2)	*Bacteria* (*Gram-negative*)

**Table 3:** Antibacterial properties of pure zeolites.

Zeolite type	Zeolite properties: particle size (μm); Si/Al ratio; pretreatment	Type of microorganism	Solution concentration (g/mL)	Best results MIC (μg/mL), *δ* (mm)	Reference
CZ	75–150; N/A; NaCl	*E. coli*	0.7	Very small Inhibition Zone	[133]
		*B. subtilis*			

ZA	<45; N/A; N/A	*B. subtilis* (*1*)	2	No significant antibacterial activity	[135]
		*S. choleraesuis* (*1*)			
	<45; N/A; N/A	*E. coli* (*1*)	5	No significant antibacterial activity	[134]
		*S. choleraesuis* (*1*)			
		*B. subtilis* (*1*)			
		*S. aureus* (*1*)			
	N/A; 1; N/A	*S. aureus* (*2*)	N/A	No significant antibacterial activity	[136]
		*E. coli* (*2*)			
	N/A; N/A; N/A	*P. gingivalis*	N/A	MIC = 16,384	[137]
		*P. intermedia*			
		*A. actinomycetemcomitans*			
		*S. sanguis*			
		*A. viscosus*			
		*S. aureus*			

ZS	N/A; high Si/Al ratio; N/A	*E. coli*	5	No significant antibacterial activity	[138]
		*P. aeruginosa*			
		*C. albicans*			

ZY	N/A; 10.2; N/A	*E. coli* (*3*)	1	MIC = 12,000	[139]
		*S. aureus* (*2*)			
	N/A; N/A; N/A	*S. aureus* (*2*)	N/A	No significant antibacterial activity	[140]
		*E. faecalis* (*1*)			
		*E. coli* (*3*)			
		*P. aeruginosa* (*1*)			
	N/A; 2.83; N/A	*E. coli*	N/A	No significant antibacterial activity	[45]
		*B. subtilis*			
		*S. cerevisiae*			
		*C. albicans*			

ZX	N/A; 1.64; N/A	*E. coli*	N/A	No significant antibacterial activity	[45]
		*B. subtilis*			
		*S. cerevisiae*			
		*C. albicans*			

**Table 4:** Antibacterial properties of Ag–zeolites.

Zeolite type	Zeolite properties: particle size (μm); Si/Al ratio; pretreatment	Type of microorganism	Solution concentration (g/mL)	Best results MIC (μg/mL), *δ* (mm)	Reference
ZA	N/A; 1; N/A	*S. aureus* (*2*)	1% (w/v) AgNO₃ solution	Percent reduction: 84%	[136]
		*E. coli* (*2*)		Percent reduction: 80%	
	N/A; ZA1 = 0.84 and ZA2 = 1.6; N/A	*S. aureus*	N/A	MIC for ZA1 = 32	[150]
		*E. coli*		MIC for ZA1 = 32	
		*P. aeruginosa*		MIC for ZA1 = 32	
		*B. cereus*		MIC for ZA1 = 16	
		*C. albicans*		MIC for ZA2 = 128	
		*C. glabrata*		MIC for ZA2 = 128	
		*A. niger*		MIC for ZA2 = 128	
		*P. vinaceum*		MIC for ZA2 = 128	
	N/A; N/A; N/A	*S. aureus*	2 wt.% silver	SZTA = 1200 CFU ZTA = 2700 CFU TA = 5500 CFU	[146]
	N/A; N/A; N/A	*P. gingivalis* (*1*)	2.5 wt% silver	MIC = 256	[137]
		*P. gingivalis* (*2*)		MIC = 512	
		*P. gingivalis* (*3*)		MIC = 512	
		*P. gingivalis* (*4*)		MIC = 256	
		*P. intermedia* (*1*)		MIC = 256	
		*A. actinomycetemcomitans* (*1*)		MIC = 512	
		*A. actinomycetemcomitans* (*2*)		MIC = 512	
		*A. actinomycetemcomitans* (*3*)		MIC = 256	
		*A. actinomycetemcomitans* (*4*)		MIC = 256	
		*A. actinomycetemcomitans* (*5*)		MIC = 256	
		*S. mutans* (*1*)		MIC = 2048	
		*S. sanguis* (*1*)		MIC = 1024	
		*A. viscosus* (*1*)		MIC = 1024	
		*S. aureus* (*3*)		MIC = 1024	

ZY	N/A; 10.2; N/A	*E. coli* (*3*) *S. aureus* (*2*) *E. coli*	1	DI water = 10 (μg/mL) Saline solution = 800 (μg/mL) DI water = 5 (μg/mL) Saline solution = 2000 (μg/mL)	[45]
	N/A; 2.83; N/A	*E. coli*	2.5	MIC = 200	[45]
		*B. subtilis*		MIC = 200	
		*S. cerevisiae*		MIC = 100	
		*C. albicans*		MIC = 100	
	N/A; ZX1 = 3.2 and ZX2 = 8; N/A	*S. aureus*	N/A	MIC for ZX1 = 32	[150]
		*E. coli*		MIC for ZX1 = 64	
		*P. aeruginosa*		MIC for ZX1 = 16	
		*B. cereus*		MIC for ZX1,ZX2 = 64	
		*C. albicans*		MIC for ZX1 = 512	
		*C. glabrata*		MIC for ZX1 = 512	
		*A. niger*		MIC for ZX1 = 512	
		*P. vinaceum*		MIC for ZX2 = 512	

ZX	N/A; 1.64; N/A	*E. coli*	2.5	MIC = 300	[45]
		*B. subtilis*		MIC = 300	
		*S. cerevisiae*		MIC = 100	
		*C. albicans*		MIC = 100	

ZS	N/A; high Si/Al ratio; N/A	*E. coli*	5	*δ* = 10	[138]
		*P. aeruginosa*		*δ* = 5	
		*C. albicans*		*δ* = 3	

EZ	N/A; N/A; N/A	*E. coli*	N/A	Percent reduction = 100%	[151]

CZ	100; N/A; oxalic acid and sodium hydroxide	*E. coli*	N/A	Percent reduction = 82%	[34]
		*S. aureus*		Percent reduction = 67.42%	

**Table 5:** Antibacterial properties of Zn–zeolites.

Zeolite type	Zeolite properties: particle size (μm); Si/Al ratio; pretreatment	Type of microorganism	Solution concentration (g/mL)	Best results MIC (μg/mL), *δ* (mm)	Reference
ZX	N/A; N/A; N/A	*E. coli*	2	500 ppm = growth decreased from 7.5 log to 4.75	[152]
		*S. aureus*		1000 ppm = growth decreased from 7.5 log to 4.75	

ZA	N/A; ZX_1_ = 3.2 and ZX_2_ = 8; N/A	*S. aureus*	N/A	MIC = 512	[150]
		*E. coli*		MIC = 512	
		*P. aeruginosa*		MIC = 1024	
		*B. cereus*		MIC = 2048	
		*C. albicans*		MIC = 2048	
		*C. glabrata*		MIC = 2048	
		*A. niger*		MIC = 512	
		*P. vinaceum*		MIC = 2048	
	N/A; Z3 = 0.84 and Z4 = 1.6; N/A	*S. aureus*	N/A	MIC = 2048	[150]
		*E. coli*		MIC = 2048	
		*P. aeruginosa*		MIC = 2048	
		*B. cereus*		MIC = 2048	
		*C. albicans*		MIC = 2048	
		*C. glabrata*		MIC = 2048	
		*A. niger*		MIC = 1024	
		*P. vinaceum*		MIC = 2048	

CZ	N/A; 63–100 mm; NaCl	*E. coli*	1	Percent reduction = 95.07%	[153]
		*S. aureus*		Percent reduction = 82.35%	

**Table 6:** Antibacterial properties of Cu–zeolites.

Zeolite type	Zeolite properties: particle size (μm); Si/Al ratio; pretreatment	Type of microorganism	Solution concentration (g/mL)	Best results MIC (μg/mL), *δ* (mm)	Reference
ZX	N/A; N/A; N/A	*E. coli*	2	1000 ppm = growth decreased from 7.5 log to 3.5	[152]
		*S. aureus*		100 ppm = growth decreased from 7.5 log to 2	
	N/A; ZX_1_ = 3.2 and ZX_2_ = 8; N/A	*S. aureus*	N/A	MIC = 1024	[137]
		*E. coli*		MIC = 256	
		*P. aeruginosa*		MIC = 512	
		*B. cereus*		MIC = 512	
		*C. albicans*		MIC = 512	
		*C. glabrata*		MIC = 256	
		*A. niger*		MIC = 512	
		*P. vinaceum*		MIC = 512	

CZ	63–100; N/A; NaCl	*E. coli*	N/A	Percent reduction = 94.9%	[67]
		*S. aureus*		Percent reduction = 87.35%	

ZA	N/A; ZA1 = 0.84 and ZA2 = 1.6; N/A	*S. aureus*	N/A	MIC = 1024	[137]
		*E. coli*		MIC = 256	
		*P. aeruginosa*		MIC = 1024	
		*B. cereus*		MIC = 1024	
		*C. albicans*		MIC = 512	
		*C. glabrata*		MIC = 512	
		*A. niger*		MIC = 512	
		*P. vinaceum*		MIC = 512	

ZY	N/A; N/A; N/A	*S. aureus* (*2*)	3.7980	*δ* = 2.2 mm	[140]
		*E. faecalis* (*1*)		*δ* = 2.25 mm	
		*E. coli* (*3*)		*δ* = 2.4 mm	
		*P. aeruginosa* (*1*)		*δ* = 2.25 mm	

**Table 7:** Antibacterial properties of Ni–zeolites.

Zeolite type	Zeolite properties: particle size (μm); Si/Al ratio; pretreatment	Type of microorganism	Solution concentration (g/mL)	Best results MIC (μg/mL), *δ* (mm)	Reference
CZ	N/A; 63–100 mm; NaCl	*E. coli*	0.01	Percent reduction = 18.48%	[153]
		*S. aureus*		Percent reduction = 9.74%	

**Table 8:** Major factors affecting the antibacterial virtues of ion-exchanged zeolites.

Factor	Antibacterial efficiency
Type of ion	Ag > Zn > Cu > Ni
Cation loading (CL)	
Solution concentration	Higher the concentration of ion-exchanged zeolite solution increases its antibacterial properties
Zeolite cation exchange capacity (CEC)	Lower the Si/Al content of zeolite antibacterial properties increase
Pretreatment of zeolite	Hydroxide solution showed more antibacterial properties than zeolite treated with oxalic acid solution
Ion release rate	Higher ion release rate results in higher antibacterial efficiency

## Data Availability

Data sharing is not applicable to this article as no new data were created or analyzed in this study.
